# Ovarian Leydig cells and neural crests

**DOI:** 10.1007/s13577-025-01210-1

**Published:** 2025-04-12

**Authors:** José-Luis Carrasco‑Juan, Olga Tapia, Miriam González‑Gómez, Abian Vega-Falcón, Sonia García‑Hernández, Alexis Rufino-Gómez, Rafael Méndez-Medina, Hugo Álvarez-Arguelles Cabrera

**Affiliations:** 1 Área de Histología, Departamento de Ciencias Médicas Básicas, Grado en Medicina, Facultad de Ciencias de La Salud, Universidad de La Laguna, C/Santa María Soledad s/n, Apartado 456, 38200 Santa Cruz de Tenerife, Islas Canarias Spain; 2 Área de Anatomía, Departamento de Ciencias Médicas Básicas, Grado en Medicina, Facultad de Ciencias de La Salud, Universidad de La Laguna, C/Santa María Soledad s/n, Apartado 456, 38200 Santa Cruz de Tenerife, Islas Canarias Spain; 3 Área de Anatomía Patológica, Departamento de Ciencias Médicas Básicas, Grado en Medicina, Facultad de Ciencias de La Salud, Universidad de La Laguna, C/Santa María Soledad s/n, Apartado 456, 38200 Santa Cruz de Tenerife, Islas Canarias Spain; 4 Instituto de Tecnologías Biomédicas, Departamento de Ciencias Médicas Básicas, Grado en Medicina, Facultad de Ciencias de La Salud, Universidad de La Laguna, C/Santa María Soledad s/n, Apartado 456, 38200 Santa Cruz de Tenerife, Islas Canarias Spain

**Keywords:** Ovarian Leydig cells, Neural crests, Mature cystic teratoma, Steroidogenic factor- 1, Androgen receptor, Class III β-tubulin

## Abstract

In our search for markers to identify and study ovarian Leydig cells, we utilized immunohistochemical techniques and visualized the results using conventional and confocal microscopy. We successfully employed steroidogenic factor- 1 (SF1), androgen receptor (AR), and class III β-tubulin as markers. SF1 and AR specifically highlighted the intraneural cell precursors of Leydig cells, which were previously identified in a published case of mature cystic teratoma of the ovary, and the adult ovarian Leydig cells. Furthermore, the transient expression of class III β-tubulin could be associated with the intraneural displacement of these precursors, cooperating in their migration to colonize the ovaries of adult women.

## Introduction

Along embryonic development, it is known that all the events are due to the consecutive expression of a series of regulatory genes. Regarding the development of the genitourinary system, and in summary (for review, see Pipreck, 2016) [1], one of the genes initially expressed is *Wt1*, whose product, WT1-KTS, regulates the formation of the genital ridges and kidneys. *Gata4* is then expressed in the anterior half of the genital ridge, and as it is gradually expressed in an anteroposterior direction the coelomic epithelium proliferates in the same direction, accompanied by the progressive loss of the subepithelial basement membrane. Subsequently, other genital ridge marker genes are expressed, such as *Sf1*, *Lhx9*, and *Emx2*. Among these, only *Sf1* is expressed in the genital ridges, and thus, the SF1 + gonadal precursor somatic cells will develop the adrenogonadal primordia. Later, when the gonads differentiate, *Sf1* is no longer expressed in the coelomic epithelium, but it is in the pre-Sertoli cells and fetal Leydig cells (FLC), and somewhat later becomes exclusively expressed in the FLC.

The gonads initially develop as sexually bipotential structures, meaning that they have the potential to become either testes or ovaries. During this early stage, SF1 is expressed in the somatic cells that serve as precursors to the gonads in both sexes [2]. However, as sexual differentiation progresses, the expression of SF1 decreases in the ovaries and remains very low until after birth. In contrast, as testicular development progresses, SF1 expression decreases in Sertoli cells but significantly increases in FLC, and it is so necessary that in the absence of SF1, Leydig cell (LC) differentiation does not occur [3].

Recently, we have shown in a mature cystic teratoma that ovarian Leydig cells (OLC) originate from a reservoir located in a plexus composed of para-aortic nerves and ganglia, a plexus that includes the celiac and mesenteric ganglia. Therefore, the origin of OLC can be traced back to the neural crests [4]. OLC are functional cells that produce androgens, which are secreted through a classic endocrine mechanism into adjacent vascular structures or via a paracrine mechanism to exert a local direct effect in areas associated with the course of the mesonephric ducts (also known as the Wolffian ducts).

Considering all the above, it is logical to think that the OLC found in this plexus must also be positive for *Sf1* expression. To test this hypothesis, we conducted immunohistochemical analyses on histological preparations belonging to the case of mature cystic ovarian teratoma that we initially used to establish its origin. To validate our findings, we will also compare them, using similar techniques, with those obtained in the study of ovaries of an adult woman with nodular OLC hyperplasia.

## Materials and methods

The studied material of mature cystic ovarian teratoma belongs to a 12-year-old Caucasian female (for details, please see Carrasco-Juan, 2024) [4]. The ovaries studied with similar techniques have nodular hyperplasia of OLC. These ovaries belong to a 70-year-old woman who underwent a total hysterectomy with bilateral adnexectomy due to atypical endometrial hyperplasia associated with endometrioid carcinoma infiltrating the endometrium (Type I).

Ethical approval for this study was obtained from the Ethics Committee of La Laguna University (Comité de Ética de la Investigación y de Bienestar Animal, CEIBA 2018 - 0289), including the dissociation of the samples from any information that could identify the patient. The authors, therefore, had no access to identifiable patient information.

### Immunohistochemistry

The histological sections obtained, 3 μm thick, were attached to silanized slides. After pretreatment to enhance labeling, sections were blocked with 3% hydrogen peroxide and then incubated with primary antibodies (10–40 min). Primary antibodies from Roche Diagnostics, F. Hoffman-La Roche Ltd., used were: anti-AR, mouse monoclonal anti-human, clone SP107, (dilution 1.7 μg/ml), catalog no. 06523838001; anti-calretinin, mouse monoclonal anti-human, clone SP65 (dilution 6 μg/ml), catalog no. 05992184001; anti-epithelial membrane antigen (EMA), mouse monoclonal anti-human, clone E29 (dilution 0.54 µg/ml), catalog no. 05878900001. The immunoreaction was based on peroxidase activity (PAP) and counterstaining with diaminobenzidine (DAB), subsequently briefly counterstained with hematoxylin, dehydrated in ethanol series, cleared in xylene, and mounted in Eukitt®. Positive and negative controls were used.

### Immunofluorescence in confocal microscopy

For double immunofluorescence, 3-μm-thick tissue sections were obtained as described above. For antigen retrieval, sections were deparaffinized and boiled for 20 min in 10 mM sodium citrate buffer (pH 6), rinsed in Tris-buffered saline (TBS, pH 7.6, 0.05 M), and were incubated with a mixture of monoclonal and polyclonal primary antibodies diluted in TBS overnight in a humid chamber and at room temperature. The rabbit polyclonal antibodies used were: anti-tyrosine hydroxylase (TH) (1/500, code no. ab112, Abcam); anti-SF1 (dilution 1/100, code no. AB217317, Abcam); anti-AR (dilution 1/100, code no. SAB5500006, Merck). The mouse monoclonal antibody used was: anti-calretinin (dilution 1/300, code no. 6B3, Swant); anti-SF1 (dilution 1/100, code no. PP-N1665 - 0 C, Perseus Proteomics); anti-class III β-tubulin (βIII-tubulin) (dilution 1/100, code no. T8660, Sigma); anti-chromogranin A (CGA), clone LK2H10, (dilution 1 μg/ml, code no. 0567056001, Roche). The following day, slides were rinsed in TBS and incubated for 1 h at room temperature in the dark with Alexa Fluor 488 goat anti-rabbit IgG (H + L) antibody (1:300, code #A11001, Invitrogen), and Cyanine3 goat anti-mouse IgG (H + L) antibody (1/300, code no. M30010, Invitrogen), for one hour at room temperature in the dark. They were rinsed in TBS and covered with Fluoromount-G with DAPI (REF: 00–4959- 52, Invitrogen from Thermo Fisher Scientific).

### Acquisition and processing of images

Images were taken with a Canon EOS 500D digital camera (Canon España S.A., Madrid) coupled to a Leica DMLS microscope (Leica Microsystems, Wetzlar, Germany). Brightness/contrast adjustments, as well as the digital reconstructions (the photo merge), were performed with the Adobe Photoshop CS5 Extended program. Fluorescence immunosignals were obtained using a laser scanning confocal imaging system (Leica TCS SP8, Leica Microsystems, Wetzlar, Germany).

## Results

**Fig. 1:**
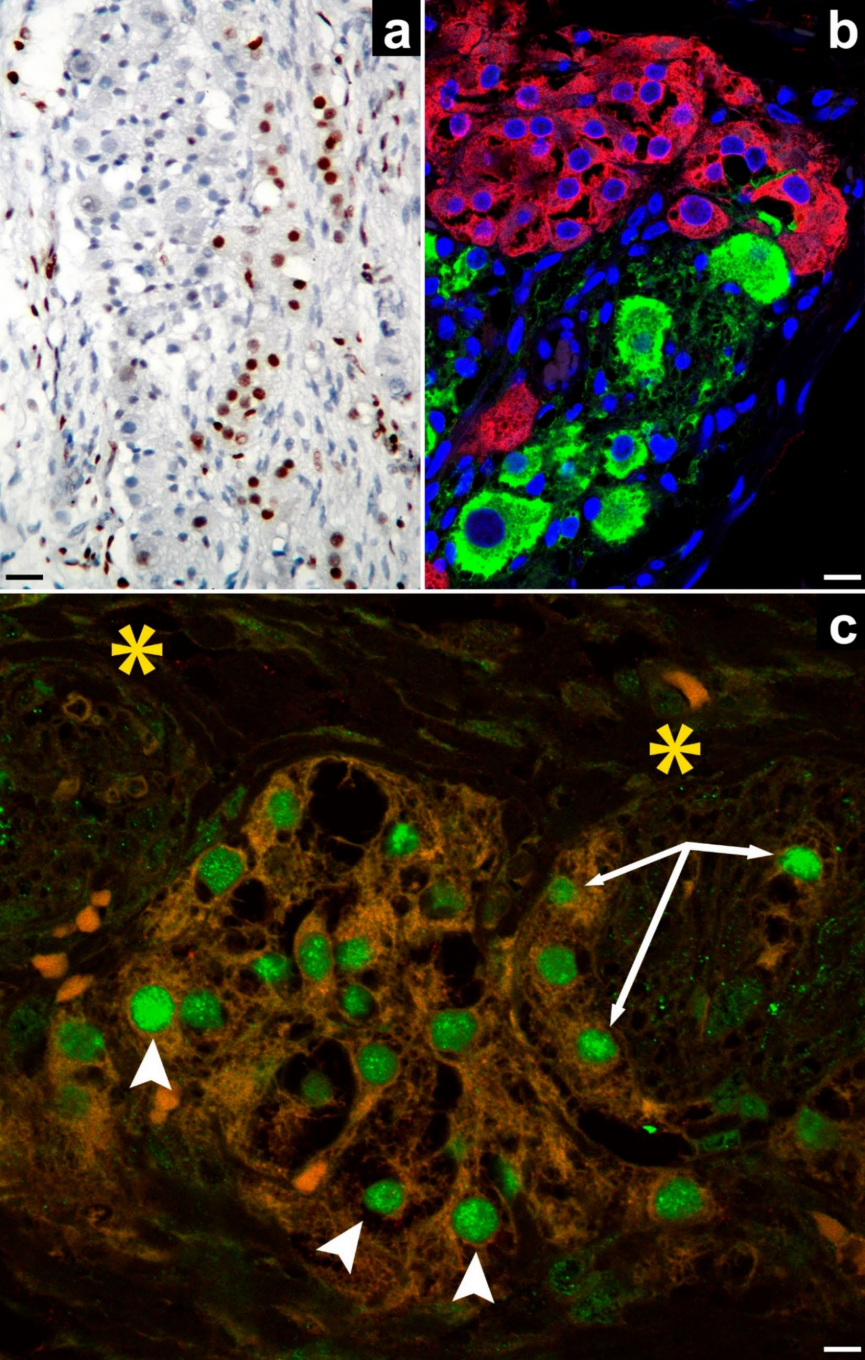
This image belongs to the teratoma. **a** It shows intraplexual LC exhibiting nuclear immunopositivity for the AR. **b** It shows that these cells display cytoplasmic and nuclear immunopositivity for calretinin (red) and that they are located within a sympathetic ganglion coexisting with ganglion neurons, which by their nature express cytoplasmic TH (green), with the nuclei stained with DAPI (blue). **c** In a somewhat more distal territory illustrates that the LC, both those within the nerves (arrows) and those located outside the nerves (arrowheads), in the stroma between two nerve silhouettes (asterisks), exhibit strong nuclear immunopositivity for SF1 (green) (scale bars: **a**, 40 µm; **b**, 15 µm; **c**, 10 µm)

In the case of the teratoma, Fig. 1a shows that the intraplexual LC (note the ganglion neurons on the left side of the image and the sympathetic vegetative nerve fascicles on the right side) exhibit nuclear immunopositivity for the AR. Figure 1b is a fusion of images showing that these LC display cytoplasmic and nuclear immunopositivity for calretinin (red) and that they are located within a sympathetic ganglion coexisting with ganglion neurons, which by their nature express cytoplasmic TH (green), with the nuclei stained with DAPI (blue). In a somewhat more distal territory, Fig. 1c shows that the LC, both those within the nerves (arrows) and those located in the stroma (arrowheads), between the two nerve silhouettes (asterisks), exhibit strong nuclear immunopositivity for SF1 (green).

**Fig. 2:**
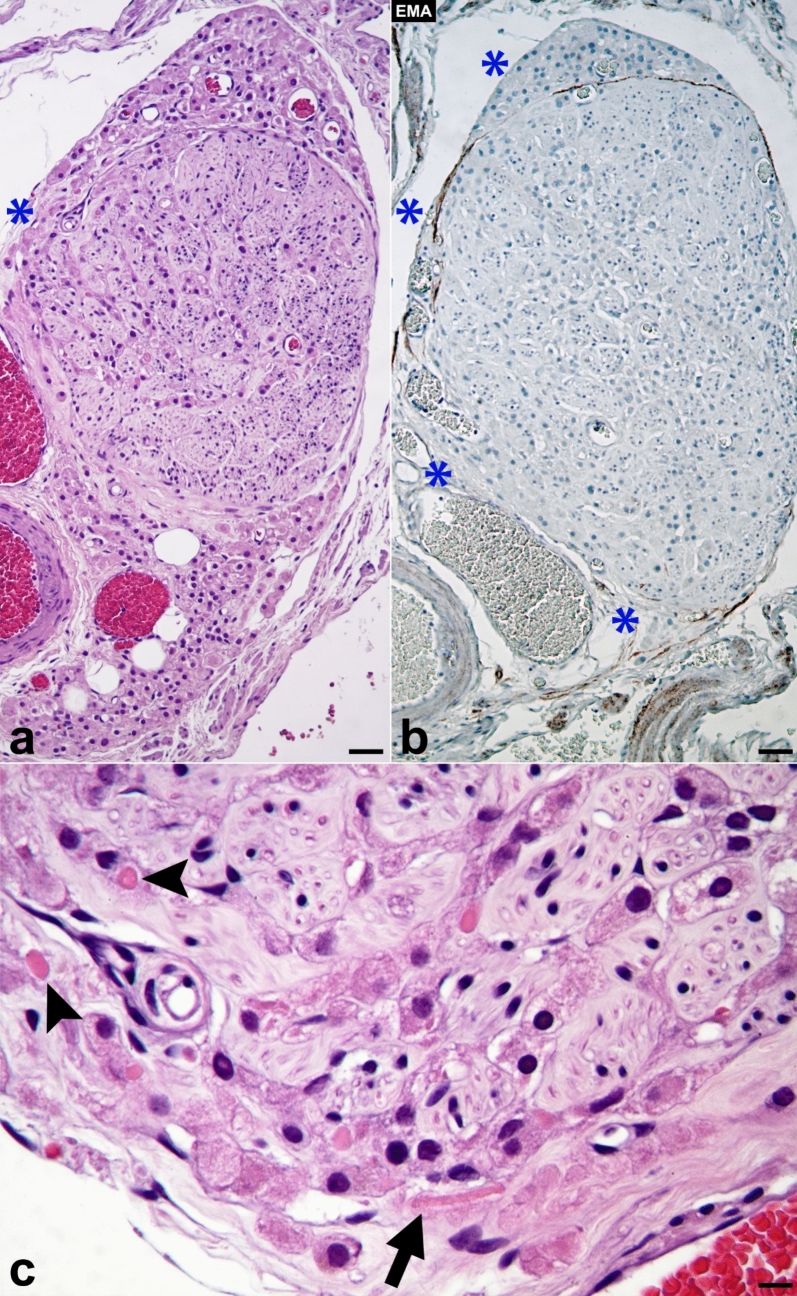
This image corresponds to the ovaries of the patient with endometrial carcinoma and nodular hyperplasia of OLC. One of the ovarian nodules is highlighted, which displays intra- and extraneural LC (**a**). The figure also illustrates the loss of the perineural sheath in several areas of the nerve (which are immuno-negative for EMA), as indicated by asterisks (**b**). With higher magnifications (**c**) and in a sector lacking the perineural sheath (2a, asterisk), LC with globoid bodies (arrowheads) and with a Reinke crystalloid (arrow) are observed (hematoxylin & eosin: **a**, **b**, × 100; **c**, × 400) (scale bars: **a**, **b**, 40 µm; **c**, 10 µm)

In the ovaries of the patient with endometrial carcinoma, Fig. 2a displays nodular hyperplasia of OLC, with one of the nodules composed of intra- and extraneural LC. Figure 2b illustrates the loss of the perineural sheath in several areas of the nerve (which are immuno-negative for EMA), as indicated by asterisks. This loss allows the LC to exit the nerve and reach the ovarian stroma. In Fig. 2c, both intra- and extraneural LC contain cytoplasmic globoid bodies, which are precursors to Reinke´s crystals (indicated by arrowheads). Additionally, another extraneural LC is shown containing a prominent Reinke´s crystalloid that measures 30 µm in length (marked by the arrow). In this sector of the ovary, highlighted with an asterisk in Fig. 2a and located at the vascular hiatuses, the perineural sheath has disappeared, facilitating the emergence of LC from the nerve and their extension into the loose connective tissue of the ovarian medulla.

**Fig. 3:**
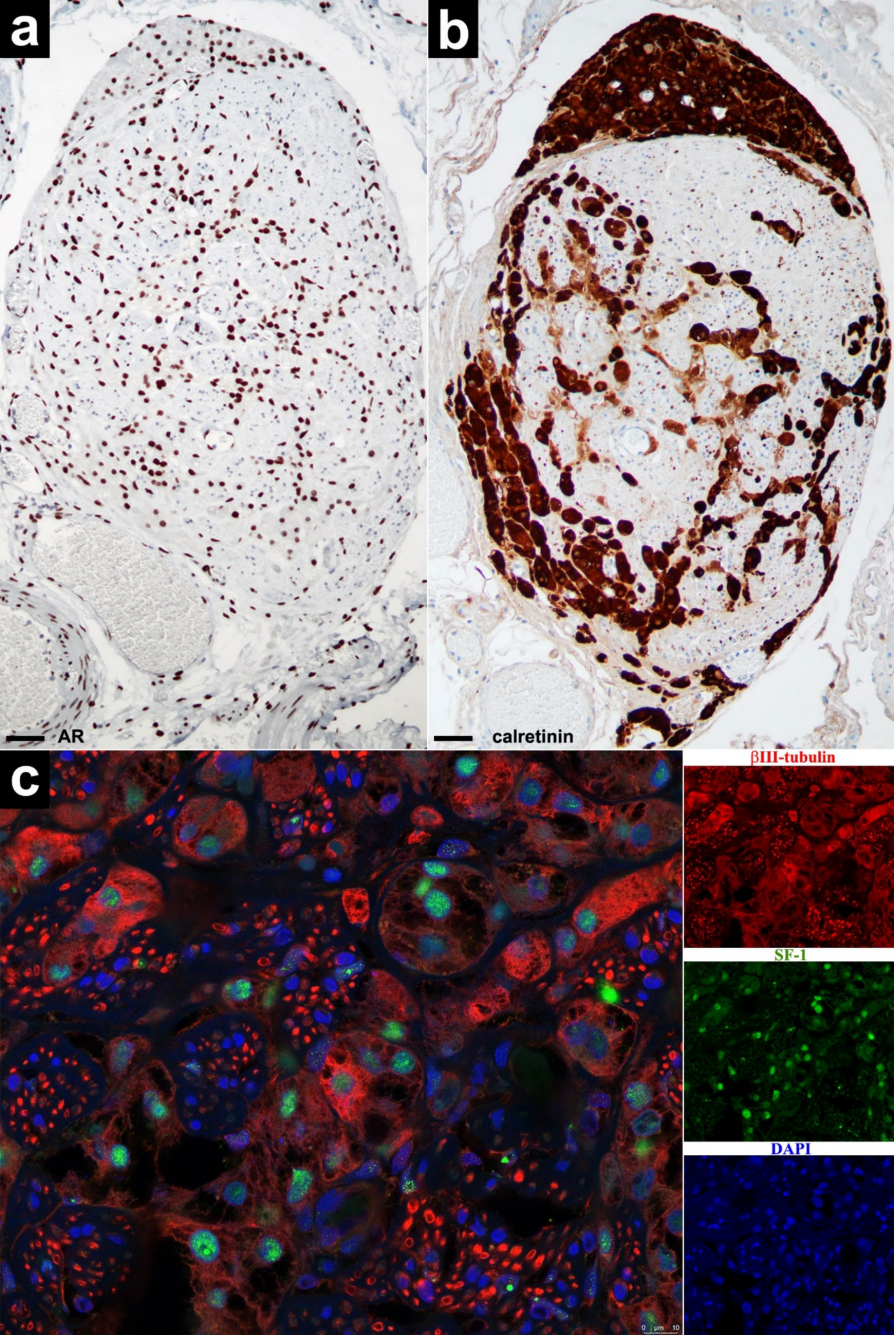
In the same node as in Fig. 2, intra- and extraneural LC show nuclear immunopositivity for AR (**a**) and nuclear and cytoplasmic immunopositivity for calretinin (**b**), but surprisingly they also exhibit variable cytoplasmic immunopositivity for βIII-tubulin (with internal positive control provided by the axons of vegetative neurons) as well as nuclear immunopositivity for SF1 (**c**) (scale bars: a, 20 µm; b, 30 µm)

In Fig. 3, we can observe the same node as in Fig. 2, which shows the immunopositivity of OLC for AR (Fig. 3a) and for calretinin (Fig. 3b). In Fig. 3c, we can appreciate the immunopositivity of the axons for βIII-tubulin, the nuclear immunopositivity of intraneural OLC for SF1, and also the variable cytoplasmic immunopositivity of some OLC for βIII-tubulin.

**Fig. 4:**
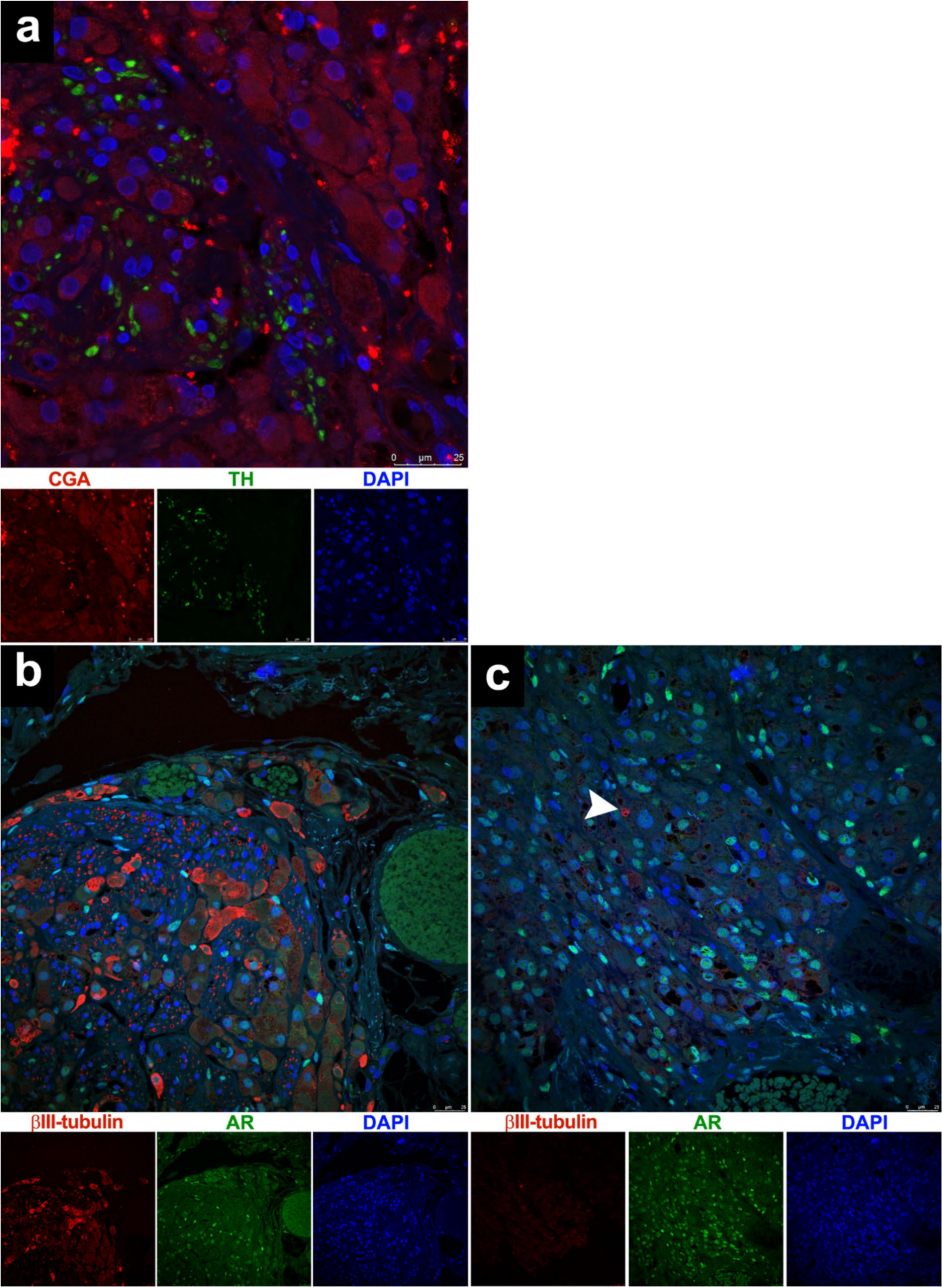
A part of the same node as shown in Figs. 2 and 3 is observed (**a**, **b**). **a** It displays intra- and extraneural OLC being negative for CGA and also TH-positive axons integrating the sympathetic autonomic nerve bundles. **b** This node contains intra- and extraneural LC exhibiting positive immunostaining for AR. It can be noted the disappearance of certain sectors of the perineural sheath and the surprising and variable cytoplasmic βIII-tubulin expression in the LC. **c** In another node, located near the ovarian cortex, it can be appreciated how these OLC have almost completely lost their expression of βIII-tubulin, which is only intensely expressed in the nerve endings (arrowhead)

In the same hilar node, Fig. 4a shows that OLC are CGA-negative and are closely associated with a sympathetic autonomic nerve that contains axons expressing tyrosine hydroxylase (TH). These cells demonstrate nuclear immunostaining for AR and many of them also express cytoplasmic βIII-tubulin in both intraneural and perineural locations (Fig. 4b). However, in the nodes closer to the ovarian cortex, these OLC nearly completely lose their expression of βIII-tubulin, with only the neuronal endings exhibit intense focal positivity (Fig. 4c, arrowhead).

## Discussion

SF1 is an orphan nuclear receptor that acts as a transcription factor, regulating cholesterol homeostasis, steroidogenesis, tissue-specific cell proliferation, and pluripotential stem cells. It plays a significant role in the development of the adrenal glands, sexual differentiation, and the functioning of LC. Thus, SF1 is expressed in steroidogenic tissues, including both fetal and adult adrenal glands, as well as in the thecal and granulosa cells of the adult ovaries, and the Sertoli and Leydig cells of developing and mature testes. Additionally, SF1 is found in other tissues such as the endometrium (where it is related to ectopic estrogen synthesis), skin, and spleen, as well as the hypothalamus and pituitary gland, where it is involved in the hypothalamic control of pituitary function [5].

SF1 is also expressed in the normal ovary, and although its levels decrease during embryogenesis, they rise again along adolescence. During fertile life, its ovarian expression is limited to the granulosa and theca cells of the follicles, and its levels increase progressively with the maturation of the follicles [6].

Although we have not found any published cases of normal adult OLC expressing SF1 immunopositivity, up to 55% of Sertoli–Leydig cell tumors are reported to be SF1 positive [7]. We have observed SF1-positive LC in both plexiform tissue of the teratoma and in the ovaries of a 70-year-old woman. In the ovaries, it is understood that SF1 regulates the expression of genes in the theca and granulosa cells that are crucial for proper ovarian function. These functions include steroidogenesis, the integration of endocrine and paracrine signals, interactions with other transcription factors, and controlling cell cycles. However, the specific roles of these SF1-positive LC in the ovaries after menopause are still to be clarified. Interestingly, their immunohistochemical characteristics, which are identical to those expressed by the plexual precursor cells of the OLC, suggest that these SF1-positive precursor cells may be the origin of both the FLC and the adult LC.

On the other hand, axons that form part of the sympathetic autonomic nerve bundles exhibit, as expected, positivity for βIII-tubulin and TH (Figs. 3c and 4a), and this is not an unusual finding. These axons belong to second neurons whose cell bodies are located in the celiac and mesenteric ganglia, and these neurons are derived from neural crests [8]. Therefore, we used the marker βIII-tubulin to demonstrate that OLC are located both intraneurally and extraneurally. However, what is most surprising in our study is that many of the intraneural and perineural OLC also show evident cytoplasmic positivity for βIII-tubulin (Figs. 3c and 4b). The only possible explanation is that when the LC are under the influence of the intraneural microenvironment, they also express βIII-tubulin to migrate toward their final destination. This positivity begins to become variable for those LC that are located in regions where the perineural sheath is becoming discontinuous and begins to receive influences from the ovarian connective tissue microenvironment. Eventually, the LC that are already located closest to the ovarian cortex microenvironment become almost completely negative for βIII-tubulin (Fig. 4c). In this regard, βIII-tubulin is considered a member of the microtubule family that is selectively expressed in nerve cells and is commonly used to identify neuronal precursor cells. However, more recent studies have discovered that this protein is also expressed in melanoblasts/melanocytes, cells that derive from the neural crests together with the neurons of the peripheral nervous system, and both cell types came from the neural plate, allowing them to express the same biomarkers [8]. Furthermore, βIII-tubulin expression has already been detected in primordial germ cells during intraneural translocation from the posterior wall of the yolk sac to the gonadal crests [9]. Therefore, our findings reinforce our belief that OLC originate from neural crests, and that they may employ this same mechanism for intraneural translocation to colonize the ovarian stroma [10].

In conclusion, we can add that:

SF1 and AR identify the intraneural and intraganglionic LC precursors as well as those LC located in the ovarian stroma throughout adult life.

A promising marker for LC that has not been previously described is βIII-tubulin. However, this marker appears to be useful only for identifying intraneural and intraganglionic LC precursors, as well as for LC at the time they leave the sympathetic autonomic nerves to populate the ovarian stroma.
